# Management of Fracture Non-union in Children: A Literature Review With Two Case Studies

**DOI:** 10.7759/cureus.97854

**Published:** 2025-11-26

**Authors:** Hani Moosavi, Carlos Curtis-Lopez, Christos Dragonas, Nicholas Birkett, Oliver Britten, Dimitrios Manoukian

**Affiliations:** 1 Trauma and Orthopaedics, Barts Health NHS Trust, London, GBR; 2 Trauma and Orthopaedics, Royal London Hospital, London, GBR; 3 Orthopaedics, Princess Alexandra Hospital, Harlow, GBR; 4 Trauma and Orthopaedics, Princess Alexandra Hospital, Harlow, GBR

**Keywords:** bone grafting, bone morphogenetic proteins, fractures, non-union, orthopaedics, paediatric, regenerative, ring fixation, trauma

## Abstract

Paediatric non-union has significant implications for patients, their families, and the wider healthcare system. These implications can be clinical, such as delayed development and poor functionality, as well as psychological with respect to their overall well-being. As such, this necessitates a greater understanding and implementation of evidence-based treatment strategies to manage and prevent paediatric non-union. This study investigates the efficacy of different treatment strategies for paediatric non-union, in the form of a literature review supplemented by an analysis of two cases which presented to our local unit(s): case 1 being a four-year-old girl with a non-union of a Milch type 2 left lateral condyle fracture and case 2 being a seven-year-old boy with a non-union of a left clavicle fracture. A comprehensive literature search, on the basis of predetermined inclusion and exclusion criteria, was initially performed capturing data from 2013 to 2023 and later refreshed to the year 2025. Articles were identified using the databases PubMed, PubMed Central, Google Scholar, ResearchGate, and ScienceDirect, with an emphasis on investigating treatment efficacy for paediatric non-union. Multiple treatment options exist for effectively addressing non-union in paediatric patients, including bone grafting, ring fixation, valgus osteotomy, and the use of bone morphogenetic proteins (BMPs). Bone grafting has been the most efficacious treatment method so far, with limited complications. Of note, injury location and characteristic morphology of the non-union are the most important in determining treatment for the said non-union. Autologous bone grafting has demonstrated the best efficacy, exhibiting up to a 100% success rate in managing complex non-unions in children with few complications.

## Introduction

Orthopaedic non-union defines the body's inability to achieve bony union, commonly described as a fracture persisting beyond nine months with no evidence of bony healing at 12 weeks [1].

Based on non-union being determined in nine months, it has a low incidence in the paediatric population, equating to approximately 0.002% [2]. Notably, some studies adopt non-conventional definitions of non-union and subsequently report an incidence of up to 10% [3]. Regardless of the definition, non-union has clinical implications for patients and healthcare professionals, as well as psychosocial implications for all involved in the patient's care.

In 2013, Mills and Simpson published a retrospective case series of 604 patients treated for non-union in the Scottish population. These were split into cohorts by age, with 180 patients under 15 years and 424 patients between 15 and 19 years being treated for non-union. In both cohorts, non-union was commonest among the male population, accounting for 60.9% and 71.9% of patients being treated in each cohort, respectively. It was also noted that non-union occurred more commonly in the upper limb versus the lower limbs, with the distal radius identified as being the most common [4].

In 2018, Zura et al., in an analysis of the paediatric fracture database in the United States, found a total non-union rate from 237,033 eligible fractures to be as low as 0.85%. This was generated from fractures across 18 different bones, but, similar to Mills and Simpson [4], reported upper limb fractures as more prone to non-union, specifically scaphoid injuries [5]. In contrast, von Rüden et al. reported a prevalence of 1.4% in patients with lateral condyle femoral fractures, though this was generated from a small cohort sample size of 18 patients [6].

Incidence and prevalence of paediatric non-union are variable in the literature, dependent on many factors including, but not limited to, the definition used for malunion, study sample size, location of injury, diagnostics, and follow-up. Nonetheless, clinical risk factors for non-union include older age within the paediatric population, male sex, patients with high BMI, and fracture severity [5,7].

Clinical symptoms indicating non-union may include difficulty with weight-bearing (84%), pain (74%), and tenderness (38%). Of radiological significance is the absence of callus formation, which may indicate oligotrophic, septic, or atrophic non-union. Despite this, some patients may in fact have hypertrophic non-union, in which callus is present but there remains no bridge between the fractured ends of the bone. Ultimately, the diagnosis is both radiological and clinical, including the persistence of fracture lines [8,9]. Other supplementary methods of diagnosing non-union involve evaluation with scoring systems such as the Radiographic Union Score for Tibia (RUST) score, as well as bone profile and endocrine blood testing [10].

Once a diagnosis of non-union is obtained, adopting the correct treatment becomes imperative. Though multiple treatment options exist, such as external fixation, resection of pseudo-arthritic tissue, autologous bone grafting, and rigid fixation, their comparative efficacy has not been well researched. This has led to therapeutic failures in the past, especially when used for inappropriate injuries [11]. Presently, bone grafting and rigid fixation are the most debated treatment modalities, with some clinicians favouring the potential of the former to promote bony union and others preferring conventional rigid fixation and osteotomisation [12].

Currently, the literature lacks a comparative and comprehensive study into all the treatment modalities for non-union [13]. Additionally, the limited evidence in this field highlights further challenges in truly identifying the most optimum treatment modality for paediatric non-union. To this end, we present two case analyses of non-union seen in our local institution(s), followed by a review of the available literature to examine the effective strategies for the management of paediatric non-union.

## Case presentation

Case 1

**Figure 1:**
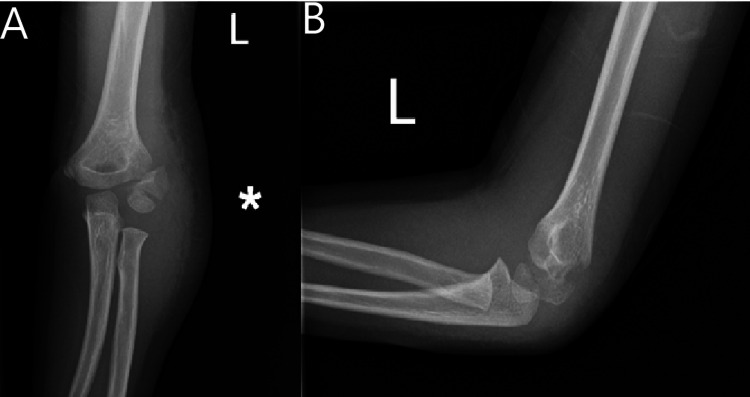
(A) Anteroposterior and (B) lateral radiographs of the left elbow showing a displaced medial condyle fracture (Case 1) The * in the figure is the local radiography department's way of indicating there is a deformity with the radiograph, in this case, to indicate the Milch type 2 fracture. It was already present on the radiograph and not added on subsequently.

A four-year-old girl presented to the emergency department following a fall onto her left elbow. Clinical examination revealed a swollen left elbow, with tenderness over the lateral joint line and condyle. Neurovascular status of the limb was unremarkable. Anteroposterior (AP) and lateral radiographs demonstrated a displaced left lateral condyle fracture (Figure 1A-1B, respectively). The patient was initially managed in the emergency department with an above-elbow plaster and was admitted for neurovascular monitoring and further imaging.

**Figure 2:**
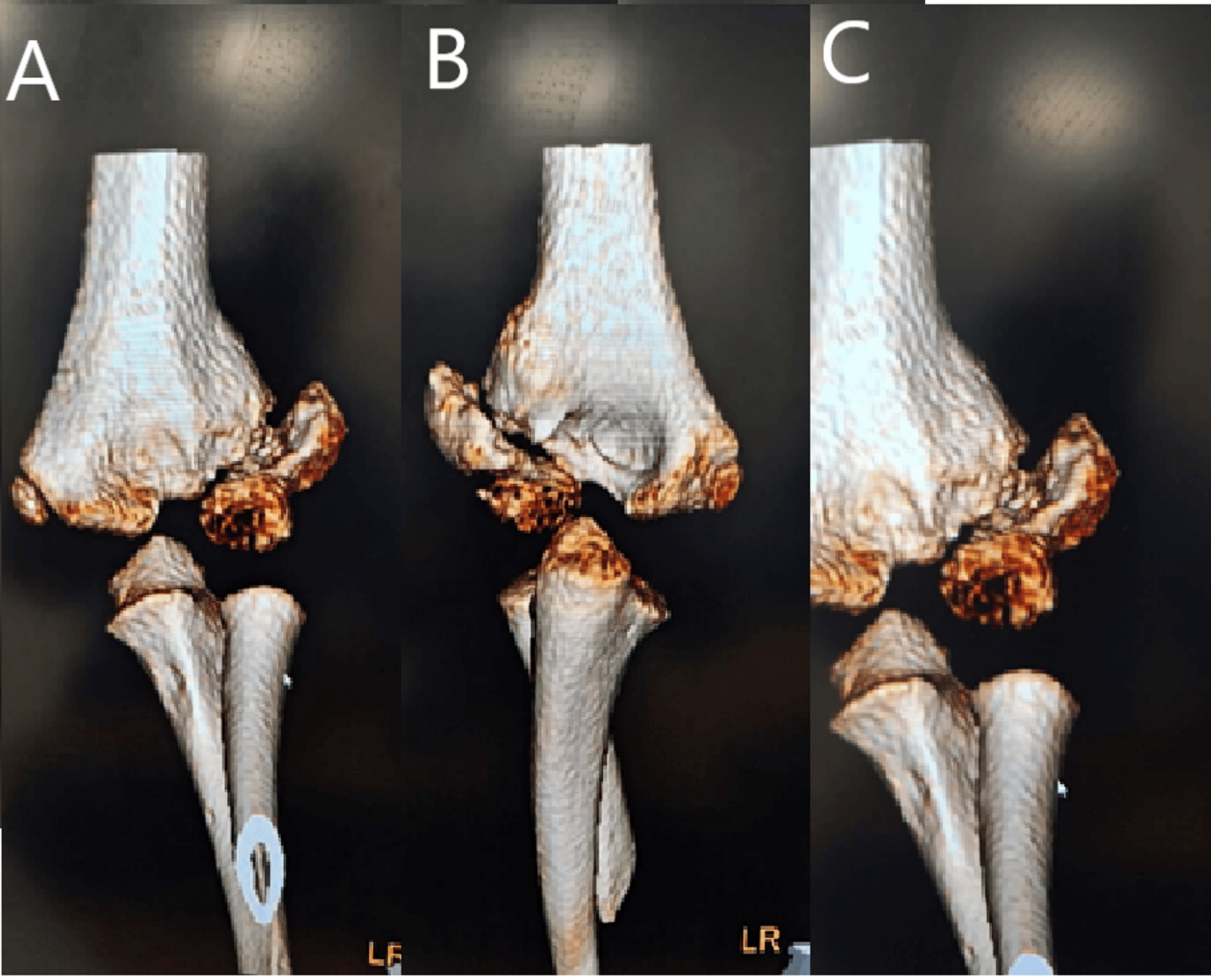
(A-C) 3D reconstructed images of the left elbow demonstrating a left medial condyle fracture (Case 1)

A computed tomography (CT) scan demonstrated a Milch type 2 lateral condyle fracture, which can be seen in the 3D reconstructed images below (Figure 2). 

**Figure 3:**
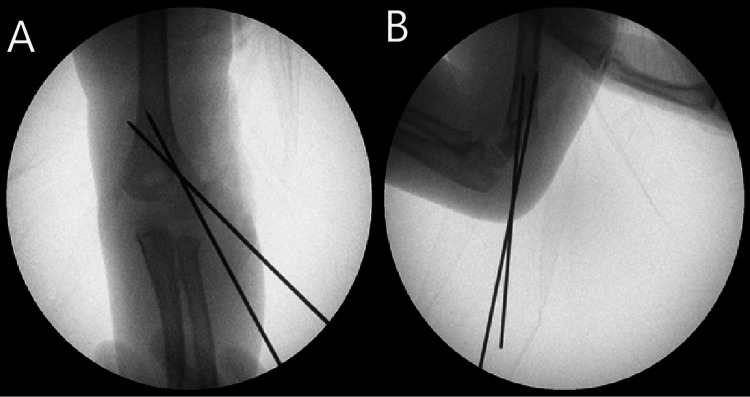
(A) Anteroposterior and (B) lateral views of the left elbow demonstrating satisfactory fracture reduction and Kirschner wire fixation (Case 1)

The patient underwent closed reduction and percutaneous pinning with Kirschner wires (K-wires). The fracture was reduced with reduction clamps, and two 1.6 mm K-wires were inserted, crossing beyond the fracture site under fluoroscopic guidance. The fracture reduction was deemed satisfactory intraoperatively (Figure 3A-3B). The patient was discharged home the same day. Mobilisation was then commenced at four weeks postoperatively following the removal of K-wires.

The patient was again reviewed at 12 weeks postoperatively where, despite returning to full range of motion and absence of pain, there was a non-tender bony prominence on the lateral aspect of her left elbow when examined. Repeat radiographs were suspicious of hypertrophic non-union, and this was confirmed on CT imaging when reviewed by our departmental paediatric orthopaedic surgeon.

Four months following her initial procedure, the elbow remained pain-free, but instability was reported in axial weight-bearing activities of the arm, such as when pushing off from a chair. At that stage, it was decided that the patient undergo revision fixation. This was achieved by open reduction, bone grafting, and cannulated screw fixation. A bone chip allograft was used to fill the fracture gap, with fixation achieved with one buried 1.6 mm K-wire and one 4 mm partially threaded cannulated screw. The elbow was immobilised for four weeks postoperatively in an above-elbow cast.

**Figure 4:**
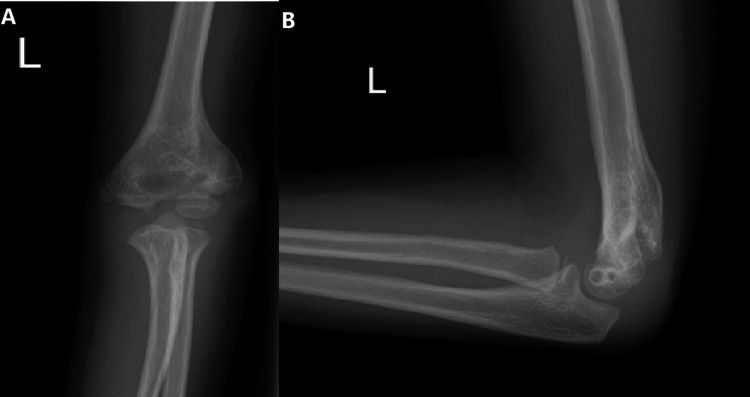
(A) Anteroposterior and (B) lateral radiographs demonstrating a union at 16 months post-injury (Case 1)

Radiographs at four weeks postoperatively showed signs of union, and clinical examination confirmed no tenderness over the lateral condyle. Further radiographs during subsequent appointments confirmed fracture union. Seven months following her revision procedure, the metalwork was removed under general anaesthesia. Intraoperatively, full fracture union was noted, and full elbow range of motion was achievable when examined under anaesthesia. The patient was discharged from the clinic 16 months following her initial injury with a full, pain-free elbow range of motion and no coronal alignment issues in comparison to the contralateral side (Figure 4A-4B).

Case 2

**Figure 5:**
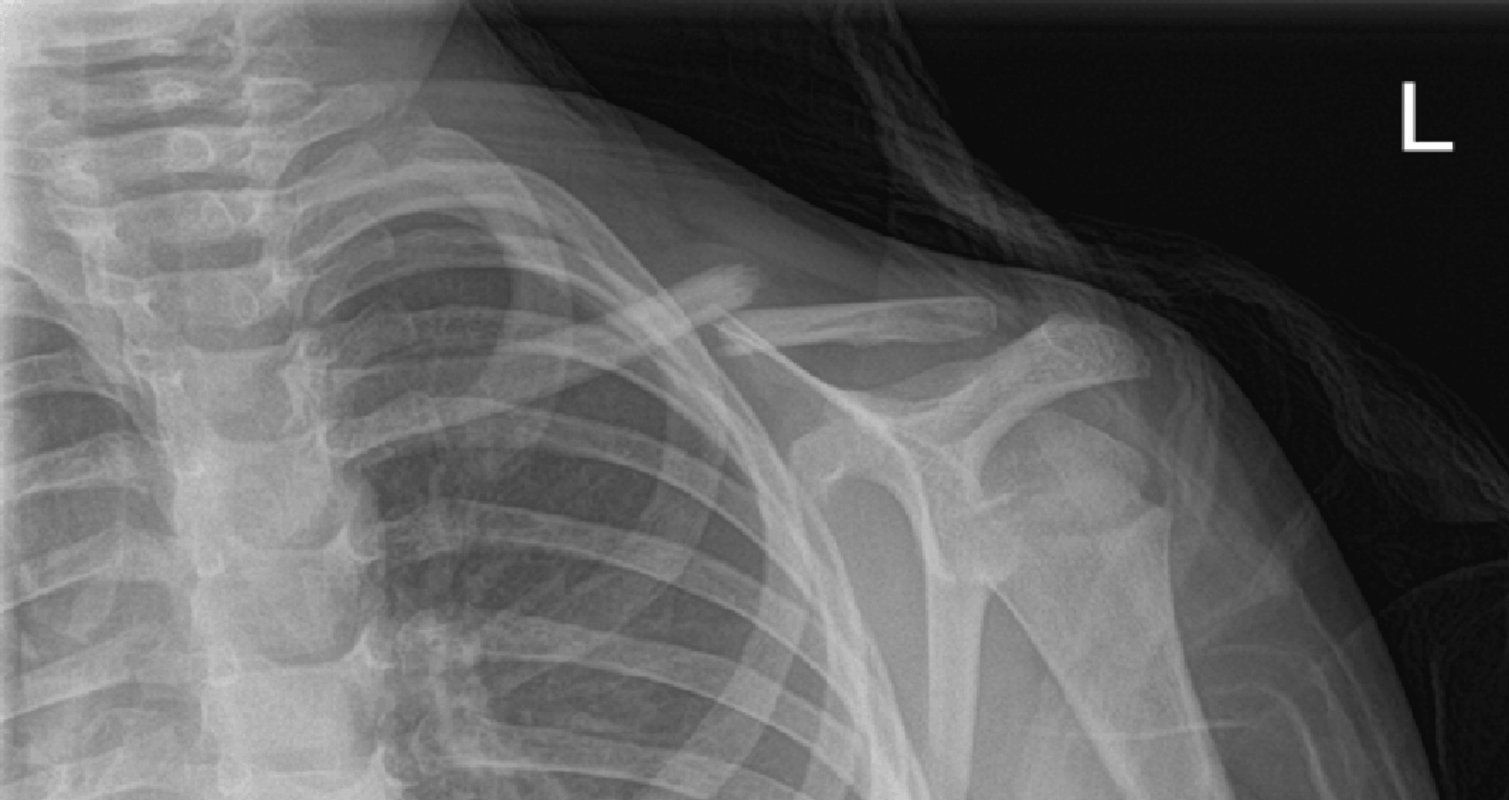
Anteroposterior radiograph demonstrating a displaced left middle third clavicle fracture (Case 2)

A seven-year-old boy presented to the emergency department following a fall onto his left shoulder. Left clavicular tenderness and an overlying palpable lump were noted on examination. Neurovascular examination of the left upper limb was unremarkable, with no threatened skin. Radiographs revealed a left clavicle fracture with complete displacement and approximately 1 cm of shortening (Figure 5). The patient was placed in a broad arm sling for comfort. Repeat radiographical imaging at the one-week clinic follow-up showed satisfactory fracture alignment and positioning.

**Figure 6:**
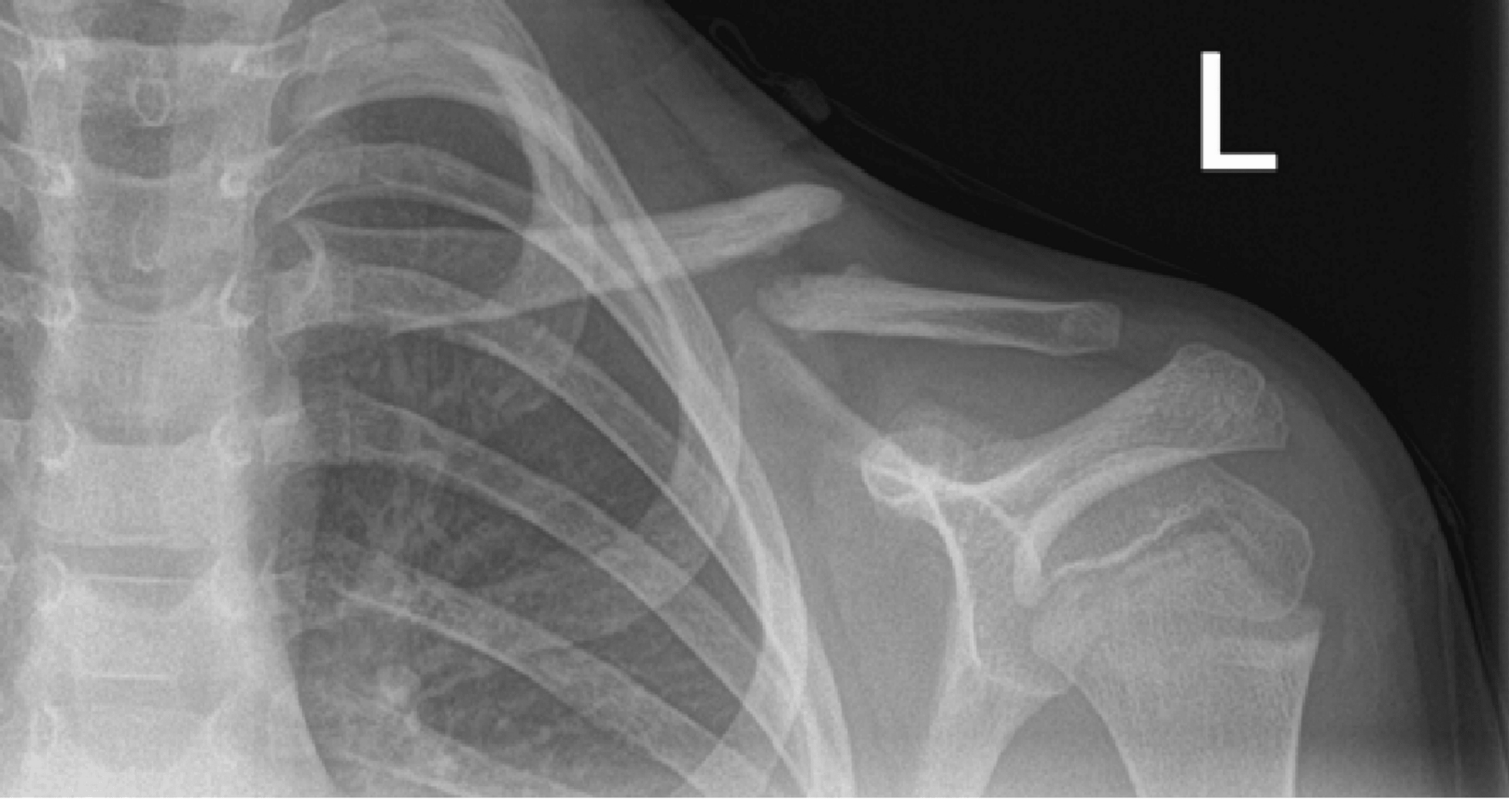
Anteroposterior radiograph demonstrating an atrophic non-union of the left clavicle (Case 2)

**Figure 7:**
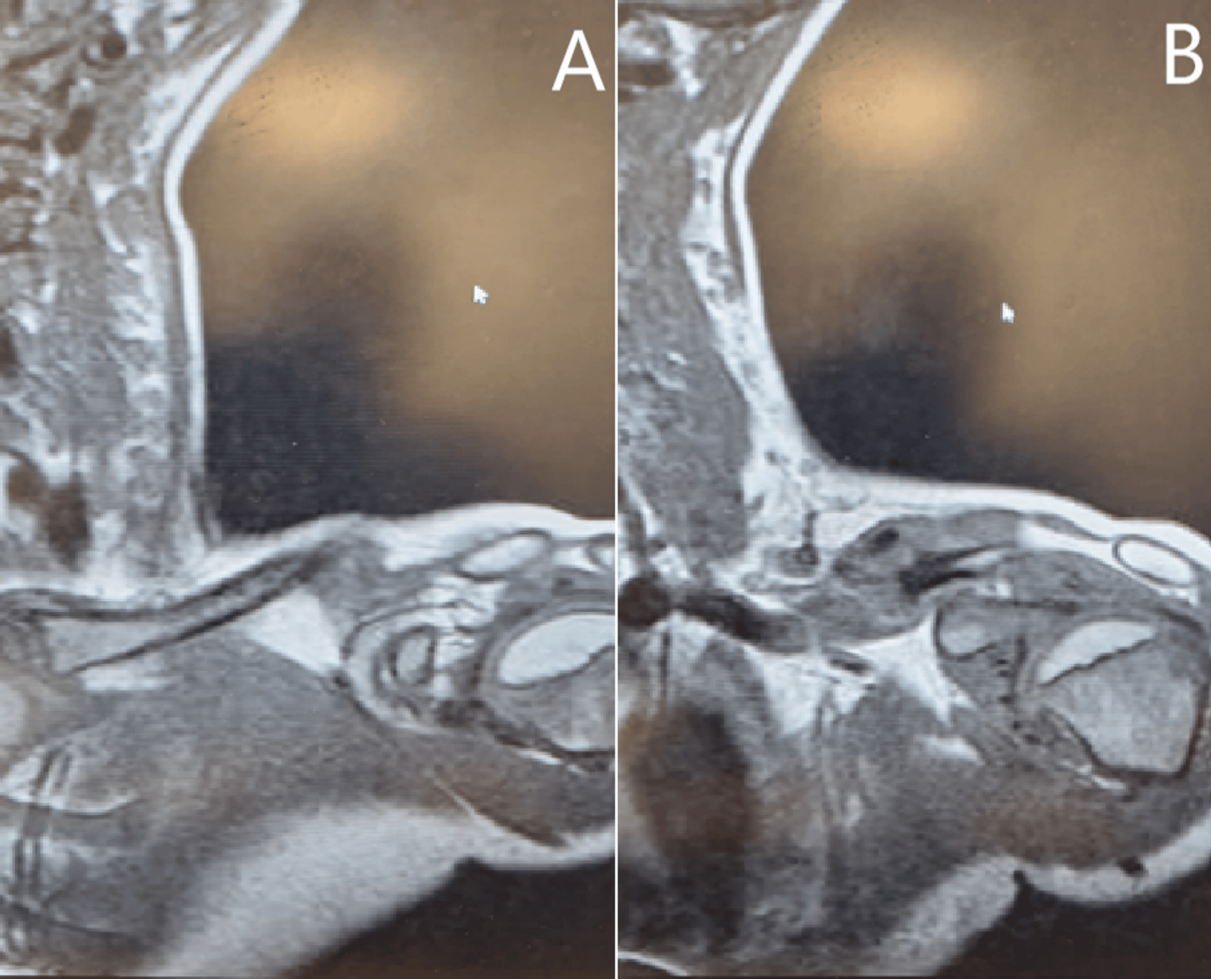
(A-B) Coronal cross-sectional imaging of the left clavicle demonstrating a non-union

Subsequent clinic reviews occurred at four, eight, and 14 weeks post-injury. Radiographs at 14 weeks revealed a persistent fracture gap and inadequate callus formation commensurate with an atrophic non-union (Figure 6). Consequently, the patient was referred to the department's paediatric orthopaedic surgeon for further assessment, with magnetic resonance imaging (MRI) (Figure 7) showing evidence of an atrophic non-union with pseudo-arthrosis formation. 

The patient and family were counselled, and based on the ongoing pain and discomfort even with simple activities and limited elevation up to 90 degrees, operative fixation was deemed the most appropriate next step. Based on a previous radiographic imaging of the clavicle, not from this admission, there is no evidence of congenital clavicular pseudoarthrosis as a differential diagnosis.

**Figure 8:**
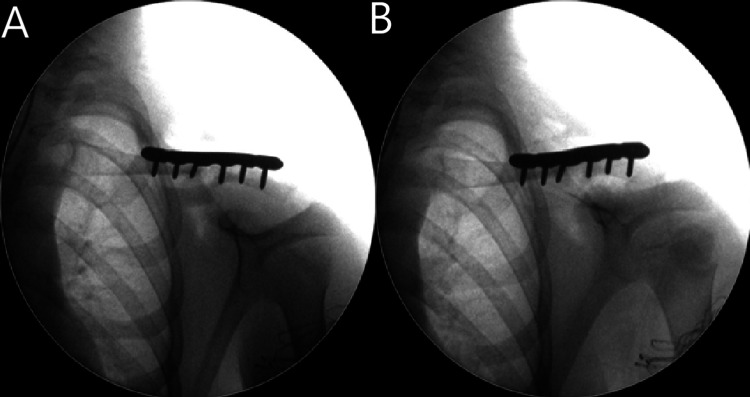
(A) An anteroposterior and (B) oblique, intraoperative radiographs showing clavicle fixation (Case 2)

Thirty weeks after the initial injury, the patient underwent open reduction and internal fixation with bone grafting. Intraoperatively, muscle interposition at the fracture site was noted. The two ends of the fracture were freshened, and reduction was achieved with a locking plate. A small amount of allograft was used to fill the defect (Figure 8A-8B).

**Figure 9:**
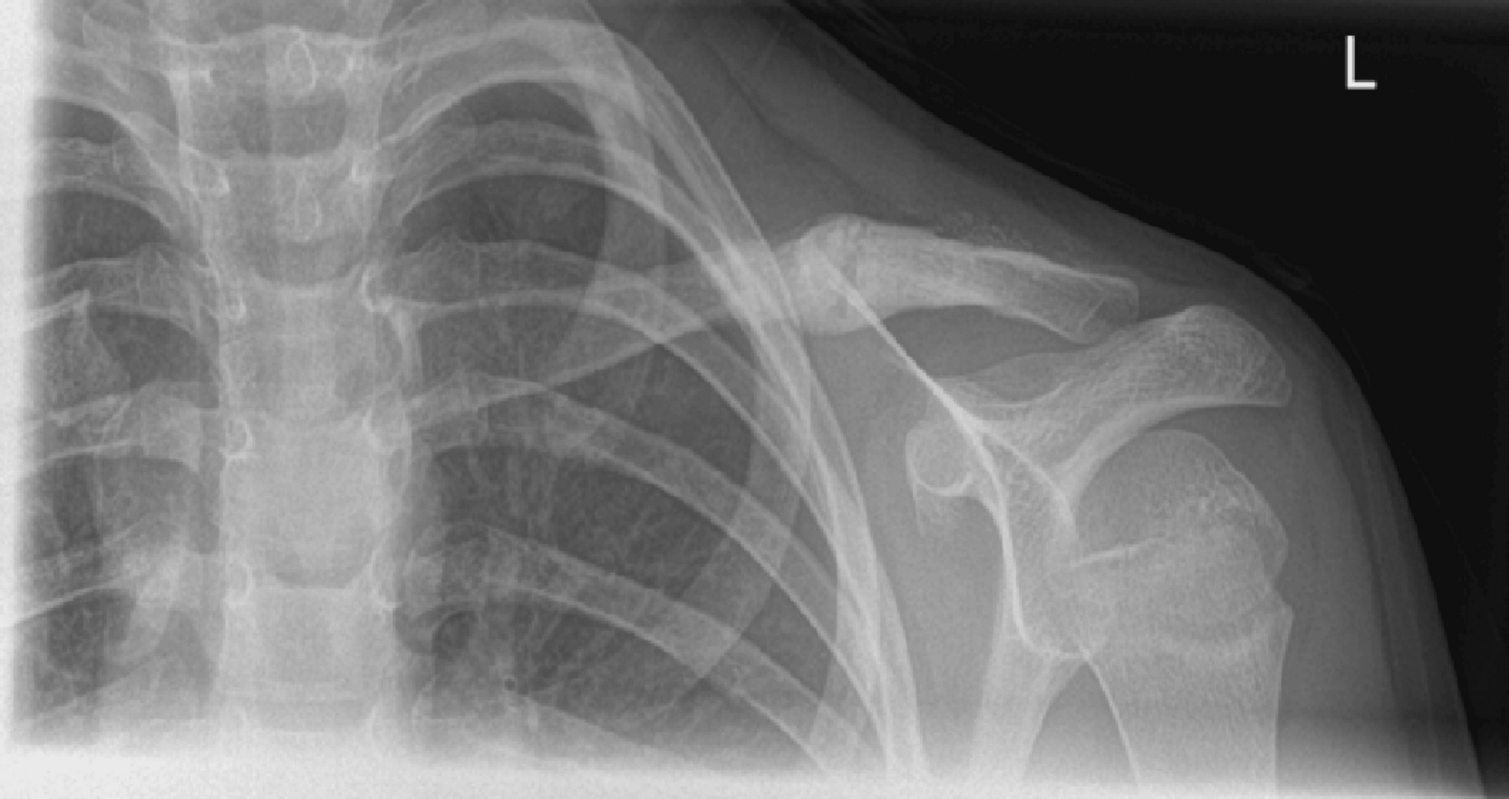
Anteroposterior radiograph demonstrating a reactive callus in the left clavicle

Following surgery, the patient's arm was immobilised for four weeks in a sling. One week postoperatively, the patient commenced with pendulum exercises and range of motion exercises of the elbow, wrists, and fingers. Four weeks postoperatively, this was upgraded to include passive range of motion exercises of the shoulder joint. At six weeks postoperatively, the sling was removed, and active range of motion with physiotherapy was commenced. At the 10-month postoperative follow-up, full range of motion was restored. Due to ongoing complaints of prominent hardware, the metalwork was removed 11 months after the operation (Figure 9).

## Discussion

Literature review

Materials and Methods

Database searches were carried out from the following five databases: PubMed, PubMed Central, Google Scholar, ResearchGate, and ScienceDirect. The initial data collection was between 2013 and 2023. The aforementioned were searched once more, accurate as of 20/04/2025, to provide contemporaneous analysis.

The inclusion criteria were as follows: English language publications, publication year 2013 or beyond, non-union described in the paediatric population only, and surgical/therapeutic management of paediatric non-union.

The exclusion criteria were as follows: non-English language publications, publications before the year 2013, full-text article unavailable, and no surgical/therapeutic management detailed for paediatric non-union.

Utilising the above inclusion and exclusion criteria, the databases were searched in accordance with the following phrases, to obtain maximal results and guide meaningful conclusions (Table 1).

**Table 1:** Key search terms for database article extraction

Search terms
'Non-union definition'
'Prevalence of non-union in children'
'Contributory factors of paediatric non-union'
'Diagnostic approaches for paediatric non-union'
'Symptomatic manifestations of paediatric non-union'
'Therapeutic modalities for non-union in paediatric patients'
'Treatment approaches for paediatric non-union'
'Intervention for non-union in children'
'Surgical Management of paediatric non-union'
'Operative management of paediatric non-union'

A summary of the obtained articles can be found in Table 2.

**Table 2:** Extracted articles following the use of search terms BMPs: bone morphogenetic proteins; PCF: posterior cervical fusion; OCF: occipito-cervical fusion; ASAMI: Association for the Study and Application of the Methods of Ilizarov; IM: intramedullary; AAI: atlanto-axial instability

Treatment	References	Sample size	Age	Clinical condition	Treatment	Results	Remarks
Bone grafting	Priano et al. [14]	2	11 and 12 years	Non-union of mid-shaft femoral fractures	Single-stage fixation approach involving a combination of intramedullary rods, plates, screws, and bone grafting	100% union rate. No significance was observed between both treatments	No complications reported
	Refai and Khalifa [15]	1	4 years	Septic non-union of the ulna	Autograft with iliac crest cancellous bone grafting and fibular strut graft	Union achieved	No complication was observed after treatment, and no additional surgical procedures were required
	Wankhade et al. [16]	539	<18 years	Donor site morbidity during fusions	PCF or OCF using autografts	Autografts: 94% union rate. Autografts + rigid internal fixation: 99% union rate	No complication was observed after treatment. No significant difference was observed between treatments
	Reintjes et al. [17]	13	15-17 years	Scaphoid non-union	Vascularised bone grafting	100% union rate. Range of motion 89%. Grip strength 92% (of the contralateral side)	No reported complications
	Koutalos et al. [18]	26	<18 years	Scaphoid non-union	Vascularised bone grafting	85% incidence of union post-treatment	Scaphoid waist fractures in 17 patients and scaphoid proximal pole fractures in 9 patients. One patient had perioperative radial nerve neuropraxia, which resolved
Ring fixation	Bakshi et al. [19]	9	<16 years	Infected both bone forearm non-union	Ilizarov frame and autologous grafting (two-stage)	100% success rate in union at 19 weeks post-op	Positive post-op functionality based on the ASAMI scale
	Magu et al. [20]	40	18 years	Long bone infected non-union	Ring fixator vs. antibiotic-coated IM nail	85% union rate. 15% complication rate	No significant difference between treatments
Valgus osteotomy	Sarkar et al. [21]	5	7-12 years	Femoral neck non-union	Valgus osteotomy combined with K-wires and screw fixation	Efficacy. Improved neck-shaft angle. Good bone density. Complications: one case of avascular necrosis	-
	Ahmad et al. [22]	1	6 years	Femoral neck non-union	Valgus osteotomy of the femur combined with K-wires, threaded pins, and plate fixation	Union achieved	-
	Sharma et al. [23]	1	3 years	Femoral neck non-union	Valgus osteotomy with solid cancellous screws and a K-wire	Patients remained asymptomatic at the two-year follow-up. Neck-shaft angle restored. No signs of avascular necrosis	-
	Eamsobhana and Keawpornsawan [24]	9	2-14 years	Non-union of the proximal femur	Valgus intertrochanteric osteotomy was performed without open reduction at the non-union site	Efficacy. 100% union rate. Functional outcomes: excellent, 2; good, 3; and fair, 4	-
BMPs	Ding et al. [25]	613	Not reported	Long limb bone non-union	BMPs with bone grafting (observation group) vs. autologous bone grafting (control group)	>90% union rate in the BMP group	-
	Xie et al. [26]	175	11 years	Patients with Down syndrome and AAI suffered from cervical spine non-union	Revision surgery using rhBMP-2	100% union rate with an average follow-up of 42.6 months	No complications reported
	Fuchs et al. [27]	5	11 years	Femoral non-union	rhBMP-2 with locking plate	Union was achieved in four out of five patients	Two complications in one patient (postoperative swelling and wound overheating, S. aureus infections)
	Cohen et al. [28]	312	≤15 years	Various cases (spinal fusion, revision, long-bone non-union)	BMP	86% union rate	2% of cases showed a direct association between BMP use and complications

Discussion

Autologous Bone Grafting

Bone grafts provide fresh osteogenic cells and naturally occurring bioactive molecules to facilitate fracture healing. Autologous bone grafting typically utilises the patient's own iliac crest as the donor site.

A case series of two young adolescent males, aged 11 and 12 years, demonstrated that bone grafting, in combination with metalwork augmentation, was successful at treating femoral midshaft non-union. Functional outcome during the final follow-up was also deemed to be satisfactory [14].

Another case report was that of a four-year-old boy, presenting with ulna septic non-union on the background of ipsilateral limb cellulitis aged four months. Union was achieved using an autologous iliac crest cancellous bone graft and a fibular strut graft. The report concluded that the success rate of autologous bone grafts was high when the harvested bone was of good quality and the recipient site was kept clean [15].

A recent meta-analysis also examined 539 patients (under 18 years old) who underwent posterior cervical fusion (PCF) or occipito-cervical fusion (OCF) procedures using autografts to reduce donor morbidity during fusions. The authors reported a high fusion rate of 94% in the paediatric population when autologous bone grafts were utilised. Moreover, when combined with rigid internal fixation, the fusion rate increased to 99%. Notably, no statistically significant differences were observed between the various fixation methods [16].

For the treatment of scaphoid non-union, vascularised bone grafting in a group of 13 adolescent patients (15-17 years) rendered a 100% union rate within an average period of 7.2 weeks, without any growth disturbances or other complications. The range of motion and grip strength achieved were 89% and 92% compared to those of the contralateral side, respectively, indicating excellent functional outcomes [17] (Table 1).

In a different study, there were findings which showed autologous bone grafting was successful at treating 85% of patients with a scaphoid non-union aged under 18 years, based on radiographic analysis and clinical examination (absence of pain, tenderness) at the six-month follow-up. The 15% failure rate was largely due to non-union occurring at the proximal pole. Despite a small sample size of 26, the results are encouraging, with only one documented radial nerve neuropraxia, which has since resolved [18].

These studies demonstrated the efficacy of bone grafting procedures, particularly those utilising autografts, exhibiting high success rates in promoting fracture union and achieving excellent functional outcomes.

Ring/Frame Fixation

The principles of ring fixation are to promote distraction histogenesis. In India, a study described the management of infected non-union of both bone forearm fractures in a paediatric population, using a two-stage approach. The first stage was a sinus excision, debridement, and Ilizarov ring fixator. The second stage, after four to seven weeks, involved autologous bone grafting to supplement the ring fixation. Union was achieved on average at 19 weeks with a good functional outcome based on the Association for the Study and Application of the Methods of Ilizarov (ASAMI) scale. This is reassuring and indicates the need to consider multimodal treatment approaches for paediatric non-union [19].

Separately, a study* *comparing ring fixator treatment with an antibiotic-coated nail fixation for infected non-union in children found an 85% union rate, with no difference between the two treatment modalities [20].

Valgus Osteotomy (VO) for Femoral Neck Non-union

VO aims to realign the femoral mechanical axis through the excision of a bony wedge from the lateral femur, with some studies demonstrating VO to have a success rate of 91% in promoting femoral neck fracture union in the adult population [21]. In the paediatric population, a case report described a six-year-old child with femoral non-union undergoing VO. The surgical procedure involved the utilisation of K-wires, threaded pins, and plate fixation, in conjunction with VO of the femur. Bony union was achieved successfully [22].

Similarly, among five femoral neck non-union patients aged 7-12 years, VO resulted in excellent union rates at both the osteotomy sites and non-union sites. The neck-shaft angle significantly improved, and the presence of good bone density indicated the remarkable bone healing potential in children (Table 1). However, a single patient presented with avascular necrosis affecting the femoral head, requiring subsequent reconstructive surgical procedures [21]. Contrastingly, in a case study of femoral neck fracture in a three-year-old, VO, along with the utilisation of K-wires and screws (to prevent plate use and a further procedure for plate removal), led to a resolution of symptoms, the complete restoration of the neck-shaft angle, and no signs of avascular necrosis in the two-year follow-up [23].

Another combination approach involving VO without open reduction demonstrated excellent results in two patients, good results in three patients, and fair results in four patients among children aged 2-14 years with non-union in the proximal femur. However, complications occurred in four patients [24] (Table 1). Although further research involving larger sample sizes and multicentre trials is necessary to validate these findings, the application of VO proved to be an effective treatment option for paediatric patients with femoral neck fractures, showing promising results with minimal complications.

Bone Morphogenetic Proteins (BMPs)

BMPs, or recombinant bone morphogenic proteins (rhBMPs), are multifunctional growth factors from the transforming growth factor beta (TGF-β) superfamily, which function to improve bone [25]. A comprehensive meta-analysis was conducted to compare BMP to autologous bone grafting (control group) for treating long limb bone non-union in 613 cases. The results demonstrated no significant differences between the groups in terms of postoperative healing rate, infection, and secondary operation rates (p>0.05), but did suggest that BMP treatment accelerates union. Notably, BMP treatment achieved success rates exceeding 90% in non-union cases. However, combining BMP with bone transplantation did not yield improved outcomes and only contributed to increased treatment costs [26].

Another synergistic treatment approach, involving rhBMP-2 and internal fixation (using a locking plate) on five femoral non-union patients (mean age of 11 years), yielded union in four out of five patients at a mean duration of 12.1 months. Only one patient experienced non-union due to deep infection, including postoperative swelling and wound overheating, potentially linked to the use of rhBMP-2, leading to surgical intervention at two and four months, respectively, due to *Staphylococcus aureus *infection in one case and spontaneous symptom regression in the other (Table 1) [27].

Contrastingly, iliac crest bone grafting and rhBMP-2 resulted in a 100% union rate in 175 cervical spine non-union patients (mean age of 11 years) comorbid with trisomy 21 and atlanto-axial instability. On follow-up after 42.6 months, all patients displayed radiographic evidence of union. However, a few postoperative complications were observed, attributable to factors such as surgeon variability, patient complexity, and missing retrospective review data [28]. Opposingly, when BMP was used for spinal fusion or revision in 312 cases of patients aged under 15 years, the overall complication rate was 21%, with only 2% of cases directly associated with complications related to BMP use. Notably, there was one case resulting in a major complication due to infection and delayed union [29].

## Conclusions

Non-union in children is overall rare. Nevertheless, delayed fracture healing can cause significant distress to the child and the family, as it essentially deprives the child of the potential to return to carefree activities and resume school and sports. Multiple treatment modalities exist to navigate and manage paediatric non-union, and in some instances, these can be merged to provide superior functional and clinical outcomes. Irrespectively, these treatments are not without risk of complications, and more research needs to be conducted in the form of comparative studies to further reinforce this hypothesis or indeed change clinical practice. 
